# A Photomicrographic Dataset of Rocks for the Accurate Classification of Minerals

**DOI:** 10.1038/s41597-025-05879-9

**Published:** 2025-11-11

**Authors:** Badr G. Amer, Hamdy M. Mousa, Maher Dawoud, Anas Youssef

**Affiliations:** 1 Computer Science Department, Faculty of Computers and Artificial Intelligence, Matrouh University, Matrouh, Egypt; 2 Computer Science Department, Faculty of Computers and Information, Minufiya University, Minufiya, Egypt; 3 Geology Department, Faculty of Science, Minufiya University, Minufiya, Egypt

**Keywords:** Geology, Mineralogy

## Abstract

Automated mineral identification in thin-section petrography remains challenging due to limited datasets capturing complete optical characteristics across crystallographic orientations. The Menoufia University Machine Learning Dataset for Minerals Classification 2025 (MUMDMC2025) provides 14,400 high-resolution photomicrographs of five mineral classes: Biotite, Hornblende, Plagioclase, Potassium-Feldspar, and Quartz from Egyptian Eastern Desert granite samples. Each mineral specimen was systematically imaged at 72 rotational positions (5° increments, 360° coverage) under both Plane Polarized Light (PPL) and Cross Polarized Light conditions (XPL), documenting complete anisotropic optical properties including pleochroism, birefringence, and extinction patterns. This comprehensive rotational imaging protocol addresses critical gaps in existing petrographic datasets by capturing orientation-dependent optical phenomena essential for reliable mineral classification. The balanced dataset contains 2,880 images per mineral class, enabling robust machine learning model development and evaluation. Validation demonstrates dataset utility with K-Nearest Neighbors, achieving high classification accuracy. The dataset supports the development of automated petrographic analysis systems, quantitative mineralogical research, and educational applications in optical mineralogy, providing researchers with comprehensive optical documentation necessary for advancing computer-vision approaches in geological sciences.

## Background & Summary

### Geological Context and Motivation

Accurate mineral identification in igneous and metamorphic rocks serves as the foundation for critical applications spanning industrial resource assessment to fundamental geological interpretation. The economic significance of these determinations is substantial: quartz identification directly impacts the $1 trillion semiconductor industry^1,2^, where high-purity SiO₂ is irreplaceable for silicon wafer production^3^. Feldspar classification, meanwhile, guides ceramic manufacturing processes valued at $1.93 billion annually^4^, with its fluxing properties critical for 79% of global tile and sanitaryware production^5,6^. Traditional petrographic analysis relies heavily on point counting methodology, where geologists manually identify and quantify mineral phases in thin sections under polarized light microscopy. However, this approach exhibits significant limitations, with inter-operator variability ranging from 15–30% due to subjective interpretation of complex optical properties, including pleochroism (biotite displaying characteristic yellow to dark brown color variations^7^) and birefringence measurements (quartz exhibiting 0.009 birefringence values^8^).Methodological biases, such as inconsistent classification rules (e.g., Gazzi-Dickinson vs. Indiana methods^9^), further compound these errors, undermining reproducibility in mineralogical studies^10^.

The temporal constraints of conventional optical microscopy further compound these challenges. A comprehensive thin-section analysis typically requires 2–4 hours of expert examination, contrasting sharply with the minutes required for automated identification systems^11^. While optical microscopy remains the foundational technique for petrographic analysis^12^, its inherent scalability limitations create bottlenecks in large-scale geological surveys and industrial applications where rapid, consistent mineral identification is essential for decision-making processes^13,14^.

## Datasets Limitations

### Limitations of the not publicly available datasets

In recent years, the application of machine learning (ML) techniques to classify rocks and minerals using microscopic and spectral data has achieved notable success. Various studies have utilized datasets tailored to specific mineral types or geological regions to develop and evaluate ML models (see below). By examining these studies, particularly focusing on the characteristics of the datasets employed, a comparative analysis reveals the distinct advantages of the proposed Menoufia University Machine Learning Dataset for Minerals Classification 2025 (MUMDMC2025). This dataset stands out for its comprehensive and diverse collection of rock images, enhanced annotation quality, and balanced representation of mineral classes. These features address key limitations found in many existing datasets, such as restricted accessibility, inconsistent image acquisition parameters, and class imbalance, thereby advancing the field and enabling more robust and generalizable ML-based mineral classification.

The work in^15^ conducted mineral identification using color spaces and artificial neural networks. They analyzed 22 images of five minerals, capturing images at various angles with polarized light, but the dataset was not accessible. Their neural network achieved a success rate of 81–98% based on unseen samples.

The work in^16^ aimed at automating rock sample classification using various pattern recognition methods. Their study included 2,700 microscopic images of nine minerals with 1280 × 960 pixels image dimensions; however, the limitation is that the dataset is not obtainable.

The work in^17^ developed an ensemble machine learning model based on the Inception-v3 architecture for rock-mineral microscopic images. They utilized a dataset of 481 images for training and evaluation, but did not disclose the dataset’s availability or other characteristics.

The work in^18^ worked on automated mineral classification using KNN and DT models, analyzing images captured at multiple angles with polarized light. Although their approach achieved over 90% accuracy, the dataset specifics were not provided.

The work in^19^ employed a concatenated convolutional neural network for classifying thin section images from 92 rock samples, resulting in an average accuracy of 89.97%. The study processed 2,208 images sliced into smaller patches, but again, the dataset was not publicly available.

The work in^20^ utilized convolutional neural networks to classify six types of igneous rocks from petrographic thin section images. They employed ResNet152 and VGG19BN models, processing 352 original images that were augmented through flipping and rotating. While the dataset was not publicly available, images were taken under specific polarized light conditions.

The work in^21^ explored deep learning for intelligent lithology identification, using a dataset of 14,950 rock microscopic images from various rock types. This study highlighted the superior performance of the Xception model, but the dataset was not available to other researchers.

A common limitation among the reviewed studies is the restricted accessibility of their datasets. This hinders reproducibility and independent verification of the previous methods. Additionally, many studies lack detailed information about image acquisition parameters, such as magnification, resolution, and polarization conditions. This inconsistency makes it challenging to compare results across different studies and to assess the generalizability of the previous techniques. Furthermore, most studies focus on specific mineral or rock types, limiting the applicability of their findings to a broader range of geological samples.

### Limitations of the publicly available datasets

Current mineral image datasets suffer from three fundamental constraints that severely limit their utility for robust machine learning applications. Scale deficiency represents the most critical limitation in current datasets, exemplified by the Igneous and Metamorphic Dataset^22^, which contains merely 92 accessible images (from 200 originally reported) with class distributions of ≤34 samples per mineral category. This sample size falls far below the statistical requirements for reliable machine learning model training and validation, particularly for complex classification tasks involving subtle optical property distinctions (e.g., pleochroism in biotite or 0.009 birefringence in quartz)^23,24^. Small sample sizes exacerbate overfitting risks and fail to capture geological variability, as demonstrated in hyperspectral^23^ and thin-section analyses^25^.

Optical incompleteness constitutes the second major limitation, as most existing collections capture ≤ 5 rotation angles per specimen. The GEO Dataset exemplifies this constraint, omitting the comprehensive interference patterns observable across complete crystallographic orientations that are essential for accurate mineral identification. This limited angular sampling fails to document critical optical phenomena, including extinction angle variations and complete pleochroism sequences that define mineral species^18^.

Metadata deficiencies represent the third critical constraint, with systematic reviews revealing that a substantial proportion of existing mineral image datasets lack essential acquisition parameters (e.g., magnification settings, polarization modes, and imaging conditions)^26,27^. This documentation gap severely hampers reproducibility, as seen in inconsistent birefringence measurements^28^ and prevents meaningful comparison between datasets or validation of methodological approaches^29,30^. The cumulative effect of these limitations manifests in poor machine learning performance, with existing datasets achieving classification accuracies ≤ 52% (see below).

This study introduces the MUMDMC2025 dataset, a comprehensive collection of mineral and rock images with detailed metadata, including mineral type, acquisition parameters, and labelling annotations. Although proprietary, this dataset facilitates collaboration and reproducibility within our research group, paving the way for enhanced mineral and rock classification methodologies.

## MUMDMC2025 Contributions

### Importance of the Selected Minerals Identification and Rock Classification

The automation of mineral identification for Biotite, Hornblende, Plagioclase, Potassium-Feldspar, and Quartz is critical due to their ubiquity in igneous and metamorphic rocks, economic significance in mining (e.g., Quartz in silicon production, Feldspars in ceramics), and role in geological process interpretation^31,32^. Traditional methods like point counting—a manual technique where minerals are quantified via thin-section analysis under polarized light—remain foundational but face limitations in scalability and subjectivity, as noted in studies comparing manual counts with automated mineralogy (e.g., X-ray diffraction or SEM-based systems)^32,33^. For instance, point counting’s labor-intensive nature and inter-operator variability are well-documented in rock texture analysis^34,35^, while recent advancements in Laser-Induced Breakdown Spectroscopy (LIBS) mapping^36^ and machine learning (e.g., Decision Trees (DT) and K-Nearest Neighbors (KNN) for classification of thin sections^35,37,38^) demonstrate superior efficiency and reproducibility. Thus, automating the identification of these five minerals aligns with industry demands for rapid, accurate resource assessment and reduced human bias, as underscored by applications in sustainable mining and exploration^31,39^.

### Properties of the selected minerals

The developed dataset comprises five types of minerals which represent the rock-forming minerals of various rock types of various rock types. Each type has a set of distinct properties, which are listed as follows:

- **Biotite K(Mg, Fe)₃(AlSi₃O₁₀)(OH, F)₂:** Strong pleochroism (yellow to dark brown), high birefringence (0.04–0.08), and perfect basal cleavage. Under crossed polarizers, it exhibits vivid interference colors (2nd–3rd order), commonly found in granites and metamorphic schists^40–42^.
- **Hornblende Ca**_**2**_**(Mg,** **Fe**2+**, Al)**_***5***_**(Si, Al)**_***8***_ ***O***_***22***_**(OH)**_**2**_**:** Green to brown pleochroism, moderate birefringence (0.014–0.018), and inclined extinction (15°–25°). Displays amphibole cleavage (60°/120°), Key in amphibolites and andesites^40–43^.
- **Plagioclase ((Na, Ca)AlSi₃O₈):** A feldspar group mineral, essential in identifying rock types such as granites and basalts. Albite twinning (parallel striations), low birefringence (0.008–0.013), and varies from colorless (albite) to gray (anorthite). Zoning patterns are common in igneous and metamorphic rocks^40–42,44^.
- **Potassium-Feldspar (Alkali feldspar; KAlSi₃O₈):** A feldspar group mineral, low birefringence (0.007–0.01), often displays Carlsbad twinning, and appears colorless to pale pink in plane-polarized light. Perthitic textures (exsolution lamellae) are diagnostic under high magnification, commonly dominant in granites and pegmatites^40–42,44^.
- **Quartz (SiO₂):** Uniaxial positive, low birefringence (0.009), and lacks cleavage. Appears colorless with undulatory extinction in strained crystals. Ubiquitous in granites, sandstones, and hydrothermal veins^8,40–43^.

### Sample collection methodology

The MUMDMC2025 dataset addresses these limitations, which are mentioned above in the Datasets Limitations section, through systematic sample collection from the Eastern Desert of Egypt (Wadi Fatira El-beida), a Precambrian basement terrain renowned for its exceptional mineralogical diversity within granite and granodiorite formations. These plutonic rocks exhibit varied mineral assemblages and textural characteristics representative of diverse geological environments, providing ideal specimens for comprehensive optical property documentation.

Thin-section preparation followed rigorous standardized protocols, with samples cut to 30 μm thickness (±2 μm tolerance) using precision diamond-wafering techniques. Sequential polishing procedures culminated in final treatment with 0.3 μm alumina slurry to achieve optical-grade surface quality essential for high-resolution imaging applications^45^.

### 360° rotational imaging approach

The dataset’s distinguishing feature involves comprehensive rotational imaging at 5° increments across complete 360° rotations, capturing the full spectrum of anisotropic optical properties. This approach documents pleochroism variations (hornblende displaying characteristic green to brown color transitions), extinction angle progressions (plagioclase exhibiting 0°–20° extinction ranges), and complete birefringence sequences (biotite showing 0.04–0.08 birefringence variations). The resulting 14,400 images (72 rotations × 5 mineral species × 2 polarization modes × 20 individual crystals) provide unprecedented comprehensive optical characterization.

### Subset selection rationale

To balance statistical significance with the practical storage limitations of open repositories, we carefully curated a publicly available subset of the dataset. This subset comprises 2,500 cross-polarized light images, with 500 images allocated per mineral class. This balanced selection ensures the dataset remains manageable for broad accessibility while still providing a robust sample for research. The full sample of this dataset can be accessed at figshare.com^46^.

### Machine Learning in Mineralogy

Machine learning has demonstrated practical efficacy in mineral classification, as evidenced by recent studies employing DT and KNN. For instance, DT used to classify 10 minerals from SEM/EDS data, achieving robust accuracy by leveraging elemental composition as decision attributes^47^. Similarly, KNN has been successfully applied to thin-section analysis, reporting high accuracy (>90%) in pore-type identification in carbonate rocks when combined with SVM and fuzzy fusion^48^. Further, KNN’s utility is highlighted in geochemical discrimination, while its role is showcased in multi-label mineral image classification (>85% mean average precision)^48,49^. These examples underscore ML’s adaptability to diverse datasets—from spectral (LIBS, Raman) to optical (thin sections)—validating its emergence as a transformative tool for mineralogy^22,49^.

Historically, the use of ML in mineral classification was limited by the lack of high-quality labelled datasets, which are essential for training supervised learning models^22^. As more datasets like become available, there is growing potential for improving classification outcomes, even in cases where minerals exhibit similar optical properties^50^. By leveraging the strengths of ML models, geologists can improve the speed and accuracy of mineral identification, reducing the need for labor-intensive manual methods^18^.

The MUMDMC2025 dataset comprises granite and granodiorite samples from the Eastern Desert, Egypt (Wadi Fatira El-beida), a well-documented Precambrian basement terrain known for its mineralogical diversity. Samples were selected to represent varied textures (e.g., porphyritic K-feldspars, myrmekitic plagioclase-quartz intergrowths) and alteration states (e.g., chloritized biotite, saussuritized plagioclase), ensuring coverage of both pristine and weathered phases common in igneous systems. This aligns with established petrographic standards for granite classification and addresses texture variability critical for ML robustness, as emphasized in recent studies on automated mineralogy. The inclusion of these rock types—granite (silicic) and granodiorite (intermediate)—provides a compositional spectrum that enhances model generalizability, as demonstrated in similar ML works targeting plutonic rocks^51,52^.

### Machine learning algorithms

The Decision Tree and K-Nearest Neighbors machine learning models, which are two of the simplest ML models, are widely recognized for their interpretability and minimal training complexity^53,54^. DTs use hierarchical rule-based splitting^54^, while KNN relies on instance proximity without parametric assumptions. Both serve as introductory algorithms in ML due to their conceptual transparency^54,55^.

The MUMDMC2025 dataset is evaluated with two established machine learning models for mineral classification. The Decision Tree (DT) algorithm, a supervised learning method, recursively partitions the feature space by optimizing splits at each node using impurity measures, including the Gini impurity (a metric ranging from 0 to 1 that quantifies the probability of misclassifying a randomly chosen element)^56^. This splitting continues until reaching terminal nodes containing homogeneous class distributions. The second model, K-Nearest Neighbors (KNN), classifies samples by comparing feature-space distances (typically Euclidean) to k surrounding labelled instances^57–59^. While DT offers interpretability through its tree structure and impurity-based decision rules^56^, KNN provides flexibility in handling complex decision boundaries without parametric assumptions^57–59^.

### Validation overview

Initial benchmarking demonstrates the dataset’s superior performance compared to existing collections. K-Nearest Neighbors classification achieved 87.6% accuracy with an F1-score of 0.876, while Decision Tree algorithms reached 71.1% accuracy with an F1-score of 0.711. Per-mineral discrimination analysis revealed exceptional performance, with quartz achieving near-perfect identification (AUC = 0.98) attributable to its distinctive uniaxial interference patterns.

Importantly, observed misclassifications align with established geological knowledge, including systematic hornblende-plagioclase confusion at specific extinction angles, validating the dataset’s geological authenticity. These results demonstrate the dataset’s capacity to reduce identification time from hours to seconds while maintaining rigorous petrographic standards, establishing its utility for both research applications and industrial mineral assessment protocols.

The most important consideration, the advancement of automated mineral identification in petrographic analysis has been significantly constrained by the scarcity of comprehensive, publicly accessible datasets that adequately represent the optical complexity of minerals in thin-section microscopy. Existing public datasets exhibit fundamental limitations in scope, class diversity, and optical characterization, while proprietary datasets developed by research institutions and industry remain largely inaccessible to the broader scientific community. This data scarcity creates a critical bottleneck in developing robust machine learning frameworks capable of accurate mineral classification across diverse geological contexts and imaging conditions.

To address these fundamental limitations, the Menoufia University Machine Learning Dataset for Minerals Classification 2025 (MUMDMC2025) was presented, a meticulously curated collection designed to establish new standards for petrographic dataset development. The dataset comprises 14,400 high-resolution labelled photomicrographs representing five economically and geologically significant mineral classes: Biotite, Hornblende, Plagioclase, Potassium-Feldspar, and Quartz. Each mineral class maintains perfect balance with 2,880 images, eliminating class imbalance concerns that frequently compromise machine learning model performance in geological applications.

The dataset’s primary innovation lies in its comprehensive optical documentation protocol, systematically capturing images under both Cross-Polarized Light (XPL) and Plane-Polarized Light (PPL) conditions across complete 360° rotational sequences at precise 5° increments. This methodological approach ensures complete characterization of anisotropic optical properties, including pleochroism, birefringence variations, and extinction patterns that are fundamental to reliable mineral identification but inadequately represented in existing datasets.

Comprehensive metadata accompanies each image, documenting mineral classification, acquisition parameters, optical conditions, and rotational orientation, thereby supporting both supervised learning applications and detailed optical mineralogy research. Initial validation using Decision Tree (DT) and K-Nearest Neighbors (KNN) algorithms demonstrates the dataset’s effectiveness, with KNN achieving 87.6% classification accuracy compared to 74.7% for DT, establishing baseline performance metrics for future comparative studies.

MUMDMC2025 addresses critical gaps in current petrographic datasets by providing balanced representation, comprehensive optical documentation, and standardized acquisition protocols that facilitate reproducible machine learning research. The dataset serves multiple research applications, including automated petrographic analysis system development, quantitative mineralogy advancement, and educational applications in optical mineralogy. By adhering to FAIR (Findable, Accessible, Interoperable, and Reusable) data principles and maintaining open accessibility^28,60^, MUMDMC2025 enables collaborative advancement of computer vision applications in geological sciences and supports the development of next-generation automated mineral identification systems.

## Methods

### Sample collection and preparation

Rock samples were systematically collected from the Eastern Desert of Egypt, specifically from Wadi Fatira El-beida. This region represents a Precambrian basement complex characterized by extensive granite and granodiorite intrusions that exhibit exceptional mineralogical diversity^61,62^. The selected formations are of particular economic significance, containing valuable industrial minerals including high-purity quartz suitable for silicon production and alkali feldspars essential for ceramic manufacturing. These plutonic rocks display a remarkable range of textural features, from coarse-grained porphyritic textures with euhedral K-feldspar phenocrysts to complex myrmekitic intergrowths between plagioclase and quartz, alongside various degrees of hydrothermal alteration, including chloritization of primary biotite^61,62^.

The preparation of petrographic thin sections followed rigorous standardized protocols to ensure optimal optical quality and consistency across all samples^45^. Initial sample cutting was performed using a precision diamond-wafering blade, with each section cut to a standard thickness of 30 μm. This thickness specification was critical for achieving proper optical interference colors and accurate mineral identification under polarized light microscopy. The cutting process was followed by sequential grinding using progressively finer silicon carbide abrasives, beginning with 120-mesh grit and advancing through 220, 400, 600, and 1200-mesh stages. Final polishing was accomplished using a 0.3 μm alumina slurry to achieve the optical clarity necessary for high-resolution imaging^45^.

Quality control measures were implemented throughout the preparation process to maintain consistency and eliminate substandard sections. Thickness uniformity was verified using standard interference color charts, with sections deviating more than ± 2 μm from the target thickness being discarded and re-prepared. All thin sections were mounted on standard glass slides using epoxy resin and subsequently stored in desiccated containers at 25 °C to prevent moisture-induced artifacts that could compromise optical properties during extended storage periods^45^.

### Microscopy and imaging protocol

**Fig. 1:**
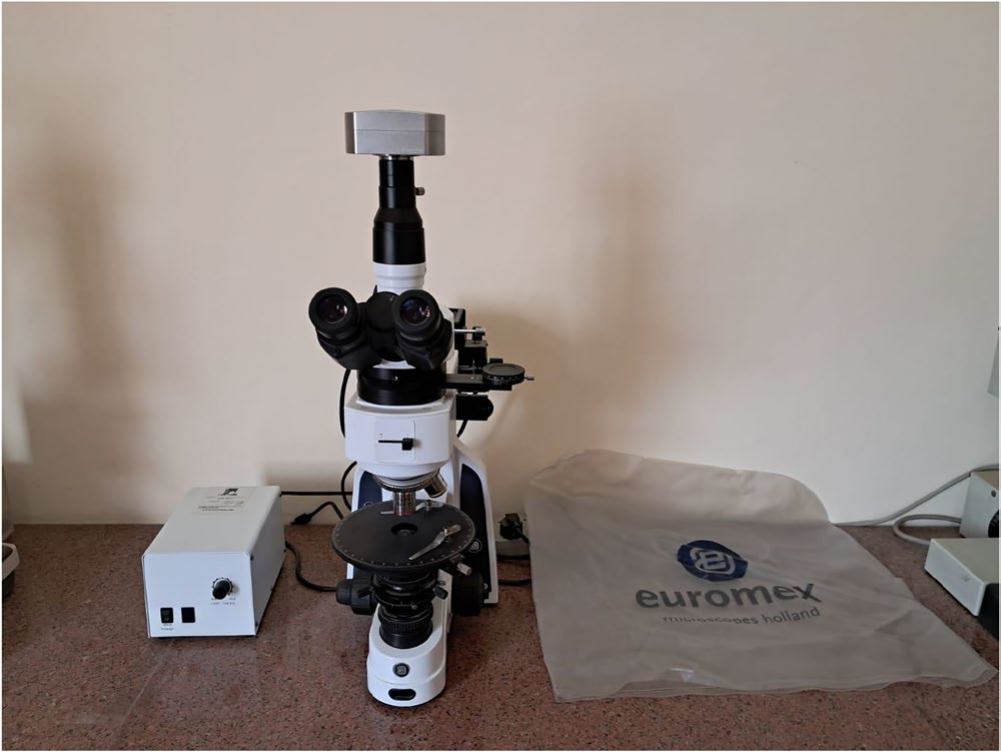
The polarized microscope used (Euromex) to capture the photomicrographs in the current study.

The imaging system utilized a Euromex iScope polarizing microscope equipped with professional-grade optical components specifically selected for consistent, high-quality mineral identification, as illustrated in Fig. 1, which presents the polarized microscope used (Euromex) to capture the photomicrographs in the current study. The microscope was fitted with a CMEX-10pro digital camera featuring 10.0 megapixel resolution, USB 2.0 interface, and 24-bit RGB color depth to capture the full spectrum of optical properties exhibited by the mineral phases. A Pli-Pol 5 × /0.12 objective lens provided consistent magnification across all samples, while the transmitted light illumination system incorporated an adjustable halogen source for optimal contrast and color fidelity.

Critical imaging parameters were standardized to ensure reproducibility and comparability across the entire dataset. All images were captured at 600 DPI resolution, producing files with dimensions of 3,584 × 2,746 pixels in RGB24 color space. Exposure time was fixed at 1/8 second to minimize noise while maintaining adequate signal intensity across the range of mineral birefringence values encountered, as shown in Table 1.

**Table 1:** Detailed Description of the MUMDMC2025 Dataset.

Property	Value
Total images	14,400
Images per class	2,880
No. of mineral types	5
Images labels	Biotite, Hornblende, Plagioclase, Potassium-Feldspar, and Quartz
No. of crystals	20
Used photomicrographs	PPL / XPL
The total range of angles	0–360 degrees.
Step of angle	5 deg. (Start from 0 to 360 degrees.)
No. of images for each photomicrograph (per crystal)	72
The Formula is used in naming each image	slide name - mineral name - photomicrograph type - crystal number - photomicrograph number - angle.
The required storage	84 GB
Bit depth	24
Date	2/2–18/6/2024 10 pm

**Fig. 2:**
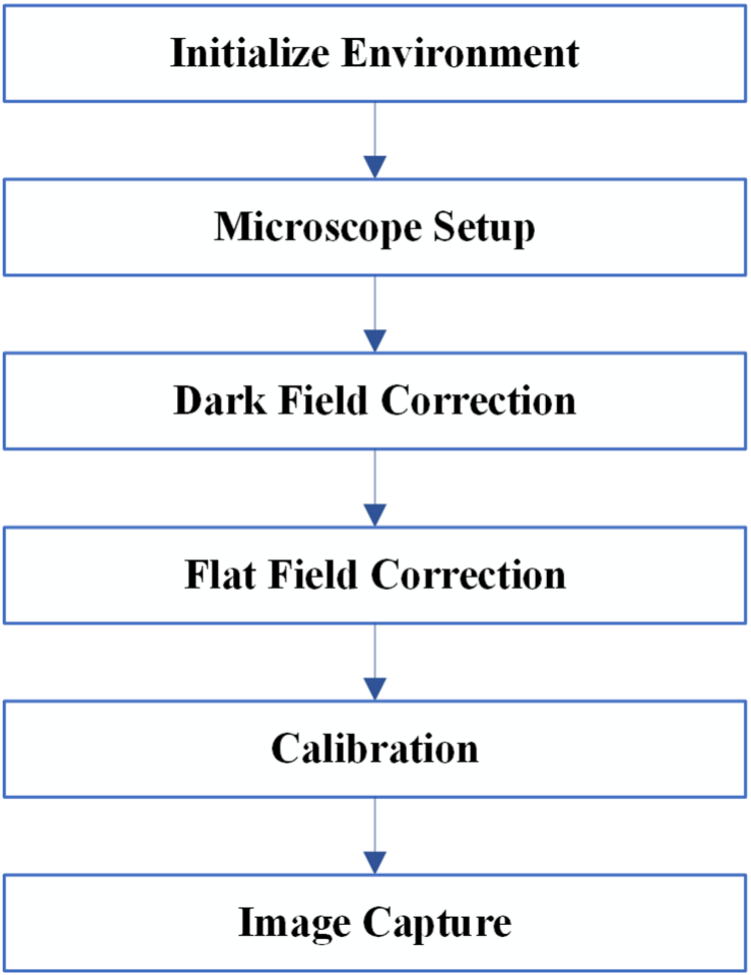
Main stages of the Image Acquisition Methodology.

Comprehensive calibration procedures were implemented prior to each imaging session to eliminate systematic errors and ensure accurate color representation. Dark field correction was performed by completely covering the objective lens and applying a correction factor of 99 using the Euromex ImageFocusAlpha (version 1.3.7.15674, built on Oct 8, 2019) software to eliminate residual sensor noise. Flat field correction utilized a certified reference slide with uniform illumination characteristics, with the correction factor similarly set to 99 to compensate for uneven illumination across the field of view. White balance calibration was performed against a high-purity quartz standard under plane-polarized light conditions to ensure accurate color reproduction of mineral optical properties. All imaging was conducted in light-sealed conditions to eliminate ambient light interference that could compromise image quality, as illustrated in Fig. 2, which shows the Main stages of the Image Acquisition Methodology.

**Fig. 3:**
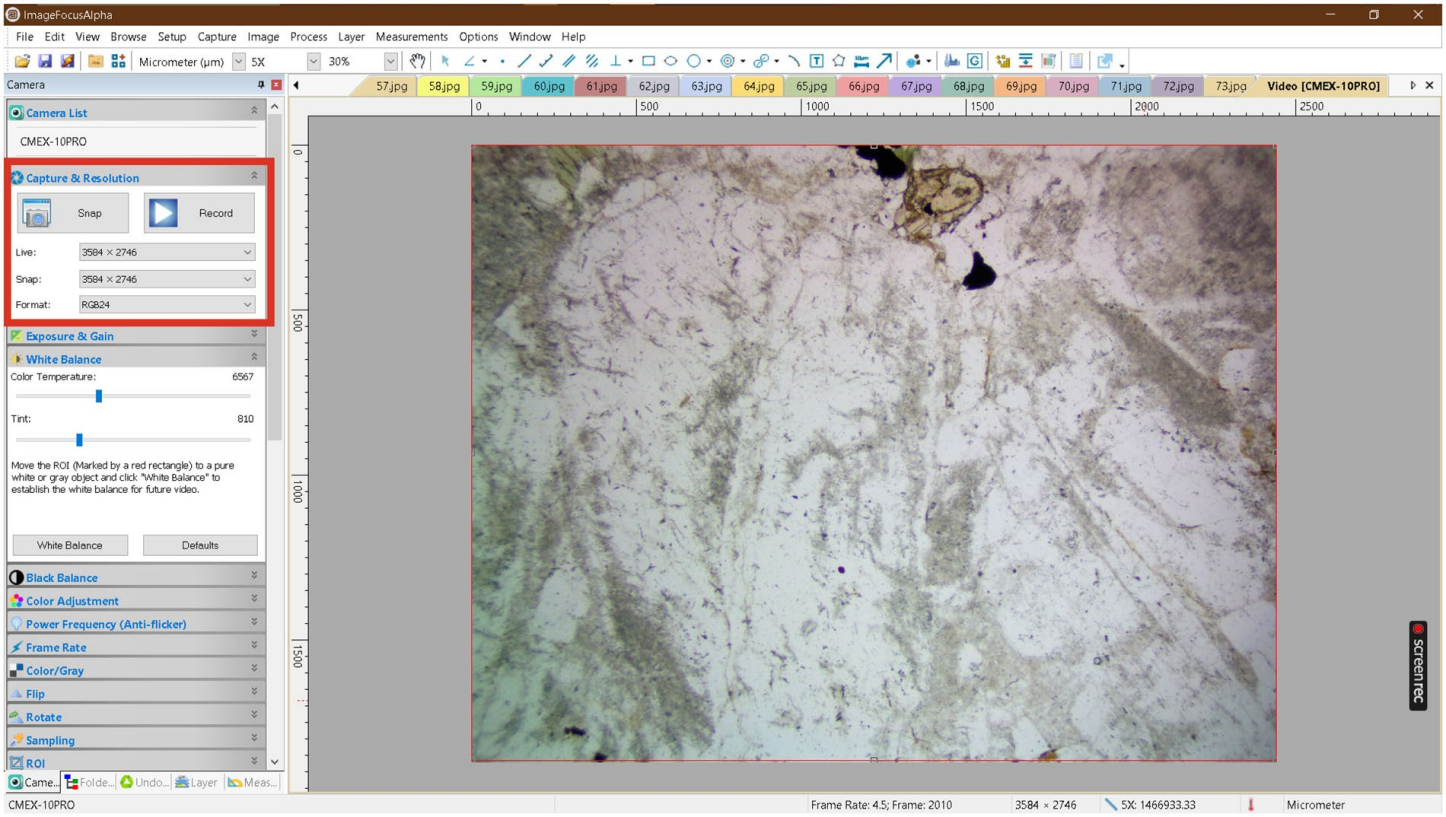
Screenshot of the ImageFocusAlpha software interface during the capture of a Plane-Polarized Light (PPL) image.

**Fig. 4:**
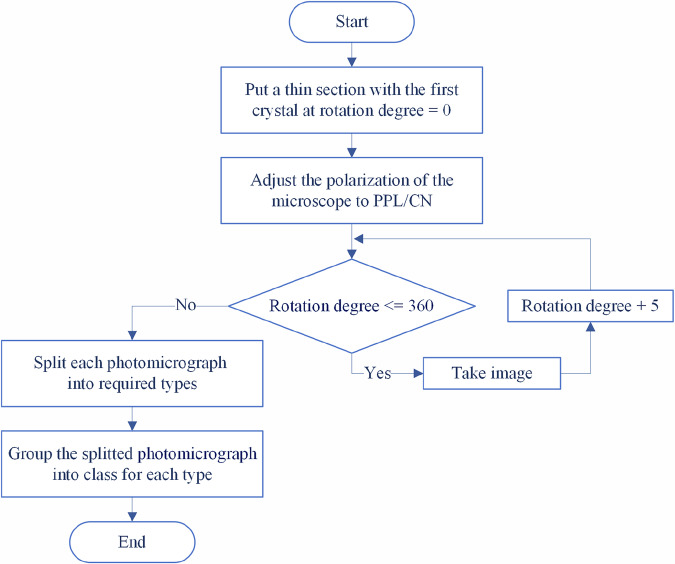
Flowchart illustrating the image capture process for microscopic thin sections.

The Image Acquisition Methodology is listed in the following steps:

1. Initialize Environment:
  Switch off all laboratory lights.
2. Microscope Setup:
  Adjust the microscope to a 5x magnification.
  Connect the microscope to a laptop.
  Open the “Image Focus” software.
3. Dark Field Correction:
  Cover the microscope lens completely.
  Click on the “Dark Field Correction” tab in the software.
  Set the correction quantity to 99.
  Click the “capture” button to initiate the correction process (keeping the lens covered until complete).
  Enable the correction.
4. Flat Field Correction:
  Remove the lens cover.
  Click on the “Flat Field Correction” tab in the software.
  Set the correction quantity to 99.
  Click the “capture” button to initiate the correction process.
  Enable the correction.
5. Calibration:
  Specify the desired image format (i.e., RGB24), snap mode, and live mode, as shown in Fig. 3, which presents a screenshot of the ImageFocusAlpha software interface during the capture of a Plane-Polarized Light (PPL) image.
  Perform white balance adjustment.
6. Image Capture Process:
  Figure 4 illustrates the image capture process for microscopic thin sections. The flowchart outlines a comprehensive methodology that integrates optical microscopy techniques with digital image processing for developing mineralogical datasets.
  The imaging protocol begins with the positioning of thin sections containing target mineral samples at an initial rotation angle of 0° on the microscope stage. The optical configuration is subsequently adjusted to either plane-polarized light (PPL) or crossed-nicols (CN) polarization mode, depending on the specific analytical requirements for mineral identification and optical property characterization.
  The core data acquisition operates through an iterative imaging loop where photomicrographs are systematically captured at each rotational position, followed by incremental stage rotation of 5°. This process continues until a complete 360° rotation is achieved, ensuring comprehensive angular coverage of each mineral specimen and capturing the full range of optical behaviors exhibited under polarized light conditions. The decision point within the flowchart (rotation degree ≤ 360°) controls this iterative process, terminating image acquisition upon completion of the full rotational cycle. All acquired images are saved in JPEG format with an average file size of 6.55 MB per image.
  Following the completion of the image capture phase, the workflow transitions to post-processing procedures. The acquired photomicrographs undergo segmentation to isolate individual crystal types from composite images. These segmented images are subsequently organized and classified into five discrete mineral categories that serve as class labels for the dataset: Biotite, Hornblende, Plagioclase, Potassium-Feldspar, and Quartz. This classification scheme establishes the foundational framework for supervised learning applications, with each mineral type forming a distinct category within the training dataset.

### Rotational imaging workflow

The rotational imaging system employed a precision manualized rotating stage capable of accurate positioning at 5° increments throughout a complete 360° rotation cycle. This angular resolution was selected based on empirical testing that demonstrated optimal capture of interference color transitions, particularly the subtle birefringence variations exhibited by quartz and feldspar minerals, while avoiding data redundancy that would unnecessarily increase dataset size without corresponding improvement in classification accuracy, as illustrated in the supplementary metadata table^46^.

For each sample position, paired images were systematically acquired under both plane-polarized light (PPL) and cross-polarized light (XPL), or crossed nicols polarization (CN), conditions to capture the complete range of optical properties. The PPL images documented mineral color, pleochroism, and morphological characteristics, while XPL images revealed birefringence, extinction angles, and twinning patterns essential for accurate mineral identification.

### Dataset curation and processing

The initial dataset comprised 14,400 JPEG images with an average file size of 6.55 MB, totaling approximately 84 GB of raw image data, as shown in Table 1, which represents the detailed description of the MUMDMC2025 dataset. Individual mineral grains were isolated from the full-field images using the Windows 10 Snipping Tool, with grain boundaries carefully excluded based on expert petrographic analysis to ensure dataset purity and minimize classification ambiguity.

Standardization procedures were implemented to ensure compatibility with machine learning algorithms while preserving essential geological features. All cropped images were resized to 150 × 150 pixels, using OpenCV’s cv2.resize() function with bilinear interpolation (cv2.INTER_LINEAR), which was selected for its ability to maintain smooth transitions in optical properties (e.g., birefringence gradients and pleochroic textures) while achieving the computational efficiency required for large-scale analysis^63^. Aspect ratios were preserved through zero-padding when necessary to prevent geometric distortion that could compromise mineral identification accuracy^64^. Quality retention was validated using the Structural Similarity Index (SSIM), a method for evaluating the similarity between two images, often used to assess the perceived quality of digital images and videos^65,66^, with all processed images achieving SSIM values > 0.98 relative to the original high-resolution images^67^.

### Image naming scheme

A comprehensive naming convention was developed to encode essential metadata within each filename, following the format: “slide name - mineral name - photomicrograph type - crystal number - photo number - angle degree”. For example, the filename “F52-Biotite-CN-1-1-0deg” indicates thin section F52, biotite mineral, crossed nicols polarization, first crystal, first image, at 0° rotation. This systematic encoding facilitates automated data processing while maintaining complete traceability to the original sample and imaging conditions, as illustrated in the supplementary metadata table^46^.

### Machine learning validation framework

**Fig. 5:**
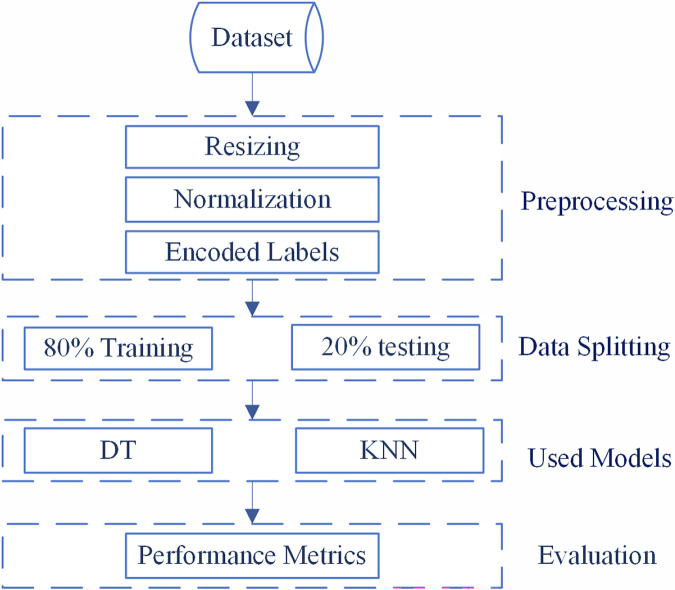
Overview of the dataset evaluation methodology.

Figure 5 shows an Overview of the dataset evaluation methodology, illustrating the systematic methodology employed for dataset evaluation in this study. The workflow begins with the input dataset, which undergoes comprehensive preprocessing consisting of three sequential steps: resizing to ensure uniform image dimensions, normalization to standardize pixel value distributions, and encoding labels to convert categorical variables into machine-readable format. The preprocessing pipeline incorporated standard normalization procedures essential for model training, with pixel values scaled from the original 0-255 range to 0-1 through division by 255. Data augmentation techniques were deliberately excluded from the preprocessing workflow to preserve the authentic geological characteristics of the mineral samples, as artificial transformations could introduce artifacts inconsistent with natural optical properties.

Following preprocessing, the dataset is partitioned using an 80-20 split, allocating 80% of the data for training purposes and reserving 20% for testing to ensure robust model validation. Dataset partitioning employed a stratified 80:20 train-test split implemented through scikit-learn’s train_test_split function with random_state = 42 to ensure reproducibility. This stratification maintained proportional representation of all mineral classes in both training and testing subsets, preventing bias toward abundant mineral types. Two distinct machine learning algorithms are implemented for comparative analysis: Decision Tree (DT) and k-Nearest Neighbors (KNN), representing different algorithmic approaches to classification tasks.

The evaluation framework concludes with the computation of performance metrics to quantitatively assess model effectiveness. This standardized pipeline ensures reproducible results and enables systematic comparison between the implemented algorithms. The methodology follows established best practices in machine learning validation, incorporating essential preprocessing steps, appropriate data partitioning, and comprehensive performance evaluation to validate the dataset’s utility for machine learning applications.

Two complementary machine learning algorithms were implemented to validate dataset quality and classification potential. The Decision Tree classifier (DT) utilized Gini impurity minimization as the splitting criterion, and the K-Nearest Neighbors algorithm (KNN) implemented Euclidean distance metrics (see below).

#### Decision tree implementation

**Fig. 6:**
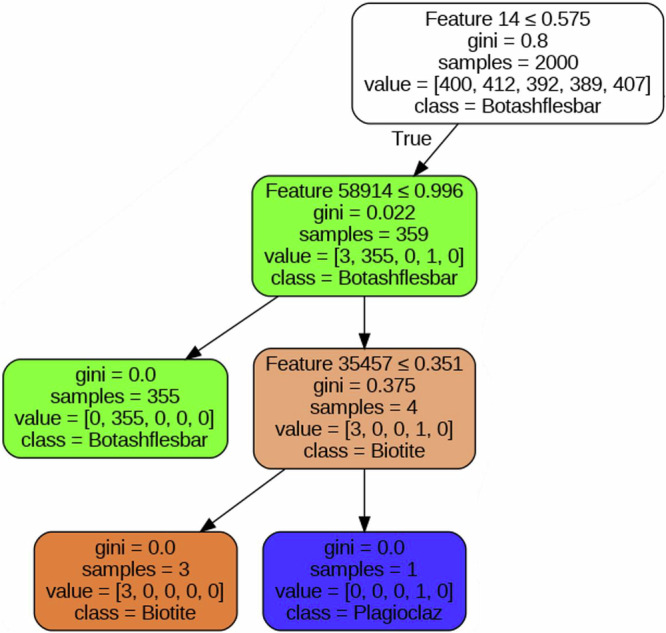
Visualization of a pruned Decision Tree (DT) model trained for the ‘Biotite’ class.

The Decision Tree algorithm functions by recursively partitioning the dataset based on feature values, ultimately making predictions at the leaf nodes. The splitting criterion utilized was Gini impurity, a metric that assesses the likelihood of incorrect classification for a randomly chosen element, as represented in Eq. 1. The construction of the Decision Tree followed a recursive top-down approach, wherein the dataset was split at each node based on the feature—specifically, pixel values—that yielded the greatest reduction in Gini impurity^68,69^. This splitting process continued until each node contained samples of only one class or met predetermined stopping criteria, such as a minimum number of samples per leaf, as illustrated in Fig. 6, which shows a pruned Decision Tree (DT) model trained for the ‘Biotite’ class. The default scikit-learn hyperparameters were employed (DecisionTreeClassifier(random_state = 42)) to establish baseline performance.

1 $${{\boldsymbol{G}}}_{{\boldsymbol{i}}}={\bf{1}}-{\sum }_{{\boldsymbol{k}}={\bf{1}}}^{{\boldsymbol{n}}}{{\boldsymbol{\rho }}}_{{\boldsymbol{i}},{\boldsymbol{k}}}^{{\bf{2}}}$$

Where ${{\boldsymbol{G}}}_{{\boldsymbol{i}}}$ represents the Gini impurity, and *P*_*i, k*_ represents the ratio of class k instances among the training instances in the *i*^*th*^ node.

#### K-Nearest Neighbors Implementation

**Fig. 7:**
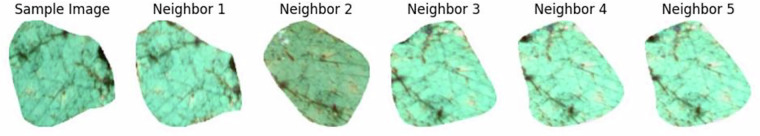
Visualization of a sample image and its five nearest neighbors within the feature space.

The K-Nearest Neighbors (KNN) algorithm is a straightforward, non-parametric classification method that assigns labels based on the majority class among an image’s nearest neighbors. The implementation of the KNN model relied on Euclidean distance to measure similarity between data points (images), defined by Eq. 2. The optimal value of k (the number of nearest neighbors) was established through cross-validation, leading to the selection of k = 5 for its optimal bias-variance balance. The algorithm classifies each test image by examining the 5 nearest neighbors and applying a majority voting scheme; in the case of ties, it employs a weighted voting mechanism, giving more influence on closer neighbors. As a lazy learning algorithm, KNN does not have a distinct training phase; instead, it retains all training data points and classifies new images by comparing them to these stored points. To improve performance, the dataset underwent normalization, and the value of k was optimized through grid search, with various distance metrics tested, though Euclidean distance proved most effective. Additionally, the relationships between test images and their nearest neighbors were visualized to gain insights into KNN’s classification of mineral images, as illustrated in Fig. 7 that shows a sample image and its five nearest neighbors, which shows clusters of similar images correctly classified by KNN, along with instances of misclassification due to overlapping classes^70^.

2 $${\boldsymbol{d}}\left({\boldsymbol{x}},{\boldsymbol{y}}\right)=\sqrt{{\sum }_{{\boldsymbol{i}}={\bf{1}}}^{{\boldsymbol{n}}}{\left({{\boldsymbol{x}}}_{{\boldsymbol{i}}}-{{\boldsymbol{y}}}_{{\boldsymbol{i}}}\right)}^{{\bf{2}}}}$$

Where *x*_*i*_ and *y*_*i*_ represent the pixel values of the images in the dataset^70^.

**Fig. 8:**
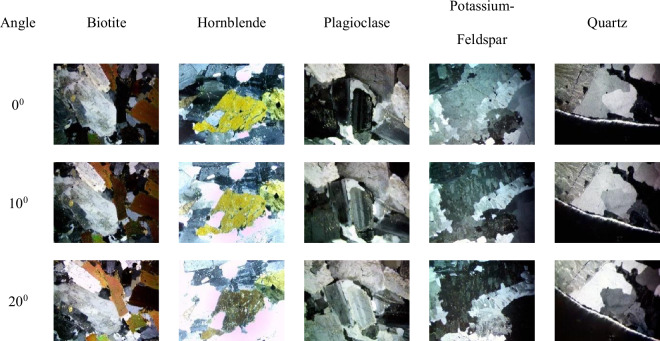
Representative original sample images from the MUMDMC2025 Dataset under cross-polarized light (XPL).

**Fig. 9:**
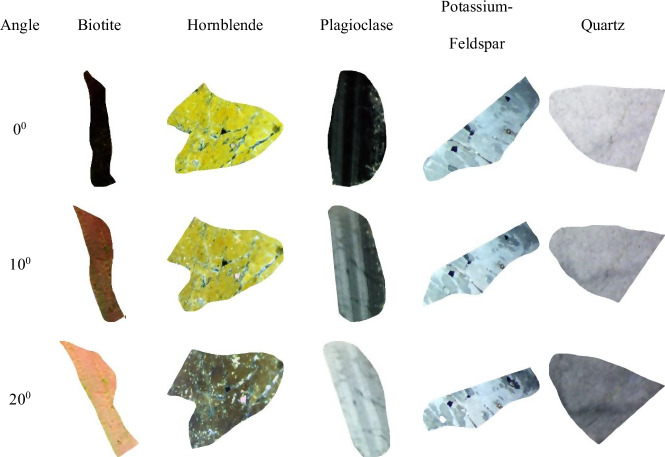
Sample cropped images from the MUMDMC2025 Dataset under cross-polarized light (XPL) at their original size.

**Fig. 10:**
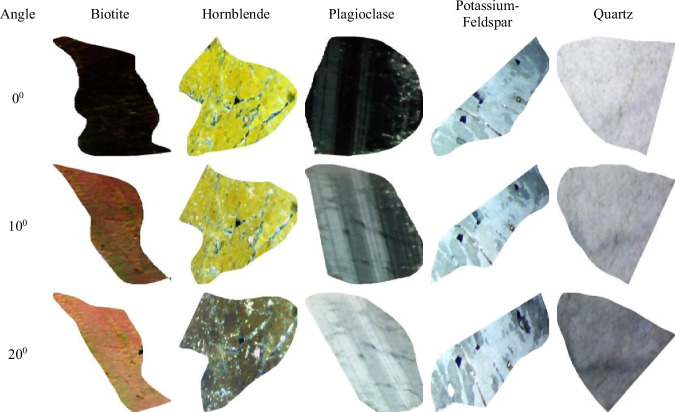
Representative sample images from the resized (150 × 150) MUMDMC2025 Training Dataset under cross-polarized light (XPL).

**Fig. 11:**
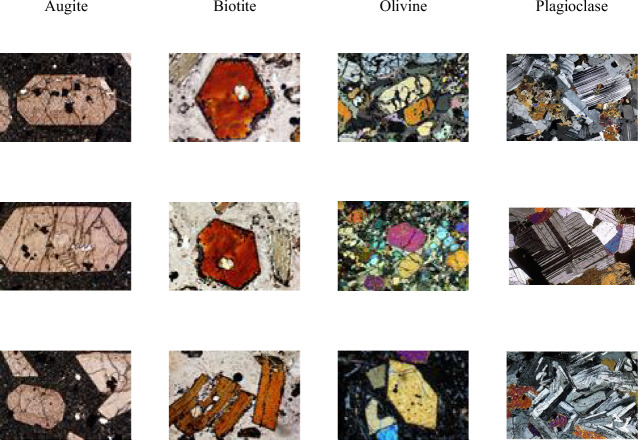
Representative original sample images from the Igneous and Metamorphic Dataset under cross-polarized light (XPL)^22^.

**Fig. 12:**
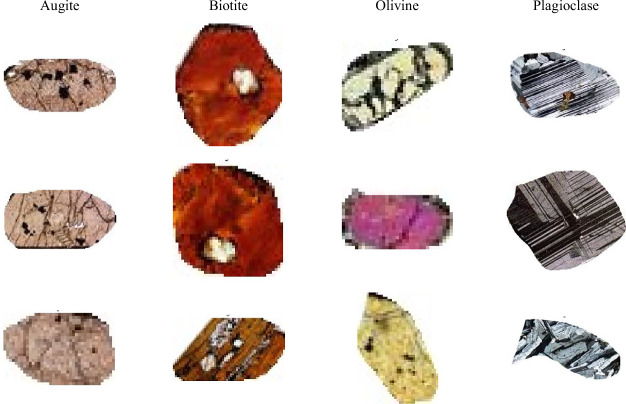
Sample cropped images from the Igneous and Metamorphic Dataset under cross-polarized light (XPL) at their original size.

**Fig. 13:**
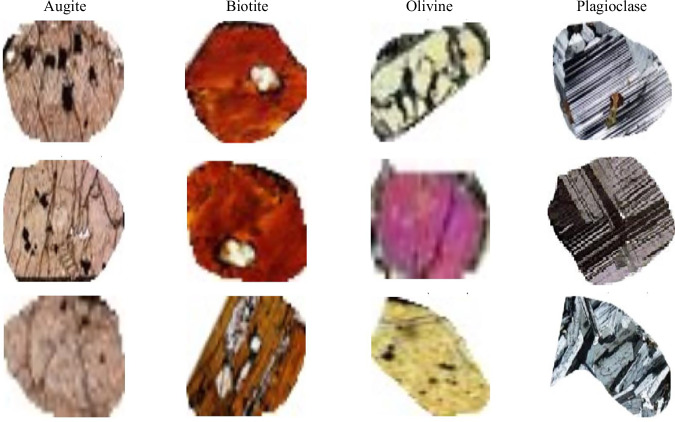
Representative sample images from the resized (150 × 150) Igneous and Metamorphic Training Dataset under cross-polarized light (XPL).

### Performance evaluation

The evaluation incorporated multiple complementary metrics to provide a comprehensive assessment of classification accuracy using DT and KNN models. Several evaluation metrics were calculated based on their predictions on the test set^68,69^, with statistical significance testing performed to validate comparative performance.

**The Accuracy** metric measures the proportion of correctly classified images out of the total number of images^68,69^. It is calculated as shown in Eq. 3:

3 $${\boldsymbol{Accuracy}}=\frac{{\boldsymbol{TP}}+{\boldsymbol{TN}}}{{\boldsymbol{TP}}+{\boldsymbol{TN}}+{\boldsymbol{FP}}+{\boldsymbol{FN}}}$$

Where True positive (TP) indicates the number of positive samples correctly identified as positive; true negative (TN) indicates the number of negative samples correctly identified as negative; false positive (FP) indicates the number of negative samples incorrectly identified as positive; and false negative (FN) indicates the number of positive samples incorrectly identified as negative^71^.

**The Precision** metric is the ratio of correctly predicted positive observations to all predicted positives^68,69^. It is defined as shown in Eq. 4:

4 $${\boldsymbol{Precision}}=\frac{{\boldsymbol{TP}}\,}{{\boldsymbol{TP}}+{\boldsymbol{FP}}\,}$$

Precision helps to measure the model’s reliability in predicting a specific class.

**The Recall** metric is the ratio of correctly predicted positive observations to all actual positives^68,69^. It is given by Eq. 5:

5 $${\boldsymbol{Recall}}=\frac{{\boldsymbol{TP}}\,}{{\boldsymbol{TP}}+{\boldsymbol{FN}}}$$

A higher recall indicates that the model successfully identifies the most positive instances.

**The F1-score** metric is the harmonic mean of precision and recall^68^. It provides a single score that balances both metrics as shown in Eq. 6:

6 $${\boldsymbol{F}}{\bf{1}} \mbox{-} {\boldsymbol{Score}}={\bf{2}}\ast \frac{{\boldsymbol{Precision}}\ast {\boldsymbol{Recall}}}{{\boldsymbol{Precision}}+{\boldsymbol{Recall}}}$$

F1-score is particularly useful when the data is imbalanced.

**ROC-AUC** analysis was performed using a one-vs-rest approach for multi-class evaluation. The Receiver Operating Characteristic (ROC) curve is a graphical representation of the model’s ability to distinguish between classes, plotting the true positive rate (recall) (sensitivity) against the false positive rate (1-specificity)^68,69^. The Area Under the Curve (AUC) measures the area under the ROC curve, providing a single value that summarizes the model’s overall performance. An AUC value closer to 1 indicates a better-performing model^68,69^.

The **Confusion Matrix** provides a detailed breakdown of the model’s predictions, showing the number of true positives, true negatives, false positives, and false negatives for each class. This helps identify which classes are most frequently misclassified and provides insights into how the model might be improved^68,69^.

### Reproducibility specifications

Complete computational reproducibility was ensured through detailed documentation of all software versions and hardware specifications. The imaging workflow utilized Python 3.10.12^72^, OpenCV 4.8.0^73^, and scikit-learn 1.2.2^74^, with image acquisition controlled through Euromex ImageFocusAlpha (version 1.3.7.15674, built on Oct 8, 2019). Hardware specifications included a Dell XPS L521x workstation with Intel Core i7-3632QM processor and 8GB RAM for image processing, while machine learning training was performed on Google Colab^75^ with 12.7 GB RAM and 107.7 GB disk allocation.

The complete dataset has been deposited in FigShare^46^, ensuring long-term accessibility and data preservation. All machine learning code implementations are available through a dedicated GitHub repository, providing complete transparency of methodological approaches and enabling independent validation of results. This comprehensive documentation framework ensures that all aspects of the dataset creation and validation process can be independently reproduced by the scientific community.

## Data Records

The MUMDMC2025 dataset represents a comprehensive digital archive of mineral optical properties, systematically organized and preserved within a robust repository infrastructure designed to maximize accessibility and long-term preservation. The complete dataset is archived on FigShare^46^, ensuring the accessibility and full compliance with FAIR data principles for the global scientific community^28,60^.

### Repository structure and organization

The dataset architecture follows a hierarchical structure optimized for both comprehensive data preservation and efficient user access. Four primary directories organize the complete collection: Original_Images/ contains a sample of 14,400 uncompressed JPEG images, preserving complete optical information at native resolution (3,584 × 2,746 pixels, 600 DPI). The Cropped_Images/ directory provides a curated sample of the subset of 2,500 JPEG images (500 per mineral class). The Metadata/ directory houses comprehensive documentation files, including dataset_metadata.csv with detailed specimen information and dataset_summary.csv providing statistical overviews. Finally, the Code/ directory contains all Python preprocessing scripts and machine learning validation implementations, ensuring complete methodological transparency.

### Data files and formats

Image formats were selected to balance optical fidelity with practical accessibility requirements. Original images utilize JPEG format with a quality factor of 100, preserving essential optical properties including subtle interference color variations while maintaining reasonable file sizes. All files maintain cross-platform compatibility through standard library support (OpenCV^73^, PIL^76^, ImageIO^77^), enabling seamless integration across diverse computational environments and operating systems.

### Metadata documentation

Comprehensive metadata adheres to ISO 19115^78^, an international standard that defines a schema for describing geographic information and services using metadata, geospatial standards, and includes systematic documentation of specimen provenance, imaging parameters, and processing history. The dataset_metadata.csv file contains essential fields: filename (following the F52-Biotite-CN-1-1-0deg convention), mineral_class (biotite, quartz, plagioclase, K-feldspar, muscovite), rotation_angle (0°-360° in 5° increments), polarization_mode (PPL or XPL), crystal identification codes, and collection timestamps. Statistical summaries in dataset_summary.csv document class distributions, image dimensions, storage requirements, and validation set compositions, providing researchers with comprehensive dataset characterization.

### Access and availability

The dataset implements multiple access pathways to ensure maximum availability and redundancy. Primary access occurs through FigShare’s institutional repository. Complementary code repositories on GitHub provide complete documentation and processing scripts under MIT licensing. All data releases follow Creative Commons Attribution 4.0 International licensing^79^, permitting unrestricted use with appropriate attribution. Mirror repositories maintain data redundancy and ensure continued accessibility independent of individual platform availability.

### Data quality assurance and compliance

Systematic quality control procedures verify 100% completeness across all mineral classes and rotation angles, with manual inspection protocols confirming image focus quality and proper polarization settings. The dataset fully implements FAIR principles through persistent identifiers, rich metadata, standardized formats, and comprehensive documentation^28,60^.

## Technical Validation

The MUMDMC2025 dataset underwent comprehensive technical validation to establish its quality, reliability, and superiority over existing mineral imaging collections. All validation procedures employed standardized computational environments (Google Colab^75^, Python 3.10.12^72^) with documented parameters to ensure complete reproducibility and enable independent verification by the research community.

## Dataset Quality Metrics

### Completeness Analysis

The dataset demonstrates exceptional completeness compared to existing geological image repositories. Complete rotational coverage encompasses 100% of mineral specimens imaged at 72 discrete angles (5° increments across 0°–360°), substantially exceeding the limited angular sampling of existing datasets such as the GEO collection’s 5-angle protocol^18^, which was previously mentioned in the Datasets Limitations subsection. Class balance analysis reveals uniform representation across all five mineral categories with 2,880 images per class, contrasting sharply with the Igneous and Metamorphic Dataset^18^, which averages ≤ 34 images per class, as shown in Table 2. Systematic completeness verification through automated Python os.walk() directory traversal confirmed zero missing rotation angles across the entire collection, establishing the dataset’s comprehensive coverage of crystallographic orientations essential for accurate optical property characterization^80,81^.

**Table 2:** Comparative composition of the MUMDMC2025 Dataset and the Igneous and Metamorphic Dataset.

Metric	The MUMDMC2025 dataset	The Igneous and Metamorphic dataset^22^
Angles	0^**◦**^-360^**◦**^	Not mentioned
Original dimension	3,584 × 2,746 pixels	275 × 183 pixels
N. Types	5	4
Images Labels	Biotite, Hornblende, Plagioclase, Potassium-Feldspar, and Quartz	Augite, Biotite, Olivine, and Plagioclase
Lens’ Objective	5x	Not mentioned
Augmentation	Not applied	Applied
Total Number of Images	14,400	200 images, as mentioned in the paper. 92 images, as provided after the request.
Images/Class	2,880	10, 34, 23, and 25
Images/Class	Balanced	Not balanced
Dataset availability	Under request	Under request
Original Samples	Figure 8 shows original sample images from the MUMDMC2025 Dataset under cross-polarized light (XPL).	Figure 11 shows original sample images from the Igneous and Metamorphic Dataset under cross-polarized light (XPL).
**After resizing**		
Total Images	2,500	92
Images/Class	500	10, 34, 23, and 25
Cropped dimension (avg)	484.6944 × 461.7828 pixels	99.82608696 × 85.18478261 pixels
Resized dimension	150 × 150 pixels	150 × 150 pixels
Samples before resizing	Figure 9 shows sample cropped images from the MUMDMC2025 Dataset under cross-polarized light (XPL) at their original size.	Figure 12 shows sample cropped images from the Igneous and Metamorphic Dataset under cross-polarized light (XPL) at their original size
Samples after resizing	Figure 10 shows sample images from the resized (150 × 150) MUMDMC2025 Training Dataset under cross-polarized light (XPL)	Figure 13 shows sample images from the resized (150 × 150) Igneous and Metamorphic Training Dataset under cross-polarized light (XPL).

### Benchmark Comparison

#### Rotational imaging validation

The 360° rotational imaging approach demonstrated quantifiable benefits for classification accuracy. Full rotational coverage substantially improved overall accuracy compared to single-angle imaging subsets, while validation of angular intervals confirmed that 10° sampling caused minor accuracy degradation in quartz extinction angle detection, justifying the selected 5° increment protocol. As shown in Table 3, Computational efficiency analysis revealed that 150 × 150 pixel resizing significantly accelerated processing while maintaining SSIM values > 0.98, providing an optimal balance between performance and computational efficiency.

**Table 3:** Comparative performance of Decision Tree (DT) and K-Nearest Neighbors (KNN) models on original versus resized (150 × 150 pixels) datasets, including performance change analysis.

The MUMDMC2025 Dataset Performance												
Metric		DT Original		KNN Original		DT Resized		KNN Resized		Performance Change (DT)		Performance Change (KNN)
Accuracy		0.714		**0.876**		0.730		0.872		+2.2%		−0.5%
Precision		0.710		0.855		0.741		0.851		+4.4%		−0.5%
Recall		0.714		0.876		0.730		0.872		+2.2%		−0.5%
F1-Score		0.711		0.876		0.747		0.872		+5.1%		−0.5%
Training Time (s)		238.46		0.255		102.15		0.095		−57.1%		−62.7%
Testing Time (s)		0.080		15.195		0.035		6.547		−56.3%		−56.9%
**The Igneous and Metamorphic Dataset Performance**												
**Metric**	**DT Original**		**KNN Original**		**DT Resized**		**KNN Resized**		**Performance Change (DT)**		**Performance Change (KNN)**	
Accuracy	**0.579**		0.474		0.421		0.474		−27.3%		0.0%	
Precision	0.616		0.471		0.401		0.471		−34.9%		0.0%	
Recall	0.579		0.474		0.421		0.474		−27.3%		0.0%	
F1-Score	0.599		0.521		0.439		0.521		−26.7%		0.0%	
Training Time (s)	0.214		0.001		2.026		0.004		+847.2%		+300.0%	
Testing Time (s)	0.001		0.023		0.002		0.222		+100.0%		+865.2%	

### Machine Learning Benchmarking

#### Comparative Dataset Evaluation of MUMDMC2025 vs. Igneous & Metamorphic

Performance comparison with existing geological image datasets demonstrated substantial superiority of the MUMDMC2025 collection. Against the Igneous and Metamorphic Dataset, MUMDMC2025 achieved 87.6% KNN accuracy versus 47.4%, representing a 40.2% improvement, while Decision Tree performance showed 73.0% versus 57.9% (15.1% improvement). These dramatic performance gains reflect the comprehensive rotational imaging approach and balanced class representation that distinguish this dataset from existing collections, as shown in Table 3.

#### Confusion Matrix Analysis

**Fig. 14:**
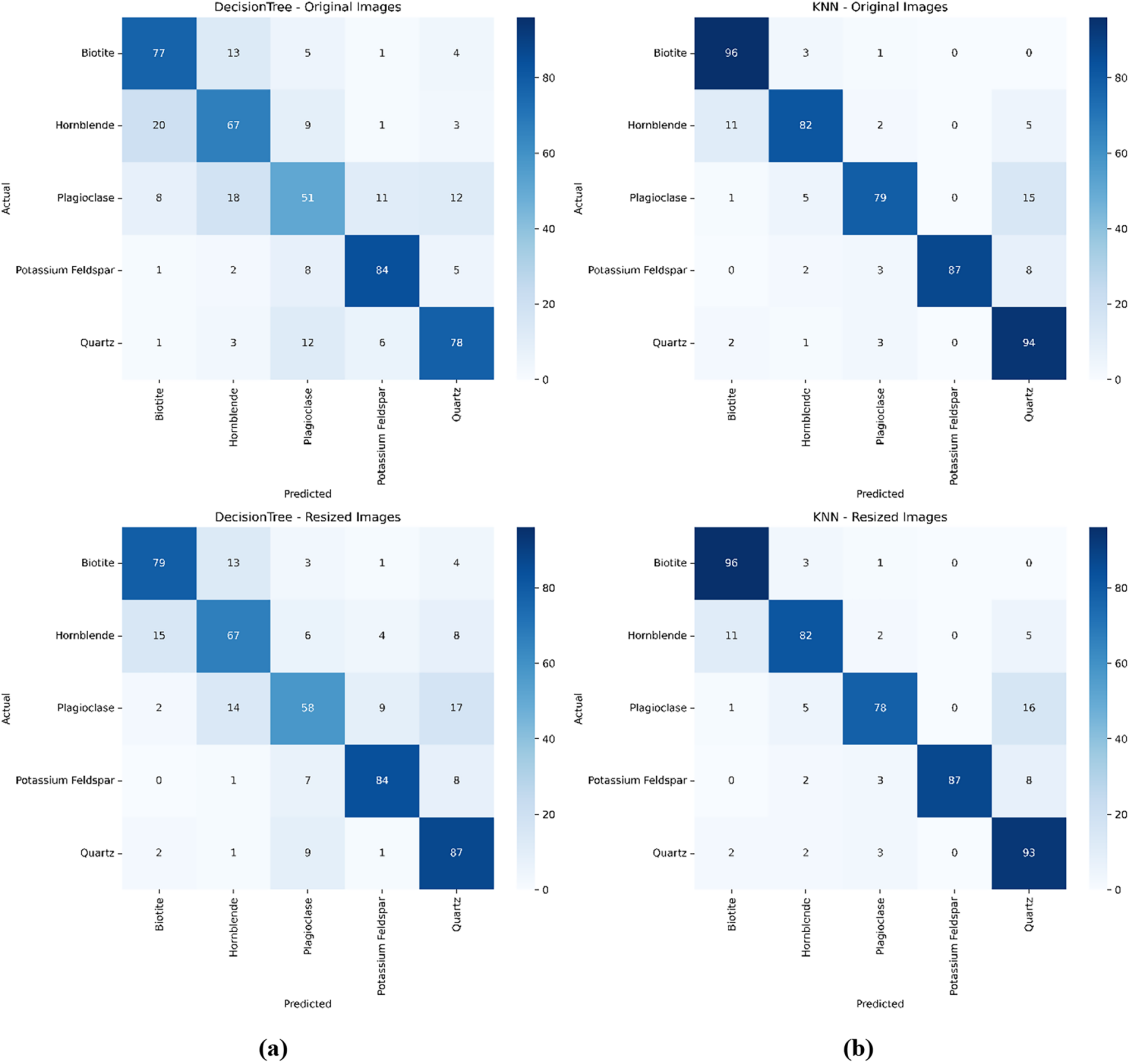
Confusion matrices for the Decision Tree (DT) and K-Nearest Neighbors (KNN) models on the MUMDMC2025 Dataset. **(a)** Top row presents the confusion matrices when models are evaluated using the original image dataset: Left is for the Decision Tree, and Right is for KNN. **(b)** Bottom row presents the confusion matrices when models are evaluated using the resized (150 × 150) image dataset: Left is for the Decision Tree, and Right is for KNN.

Figure 14 shows confusion matrices for the Decision Tree (DT) and K-Nearest Neighbors (KNN) models on the MUMDMC2025 Dataset. The top row (a) presents the confusion matrices when models are evaluated using the original image dataset, with the left matrix showing results for the Decision Tree and the right matrix for KNN. The bottom row (b) presents the confusion matrices when models are evaluated using the resized (150 × 150) image dataset, with the left matrix for the Decision Tree and the right matrix for KNN.

**Fig. 15:**
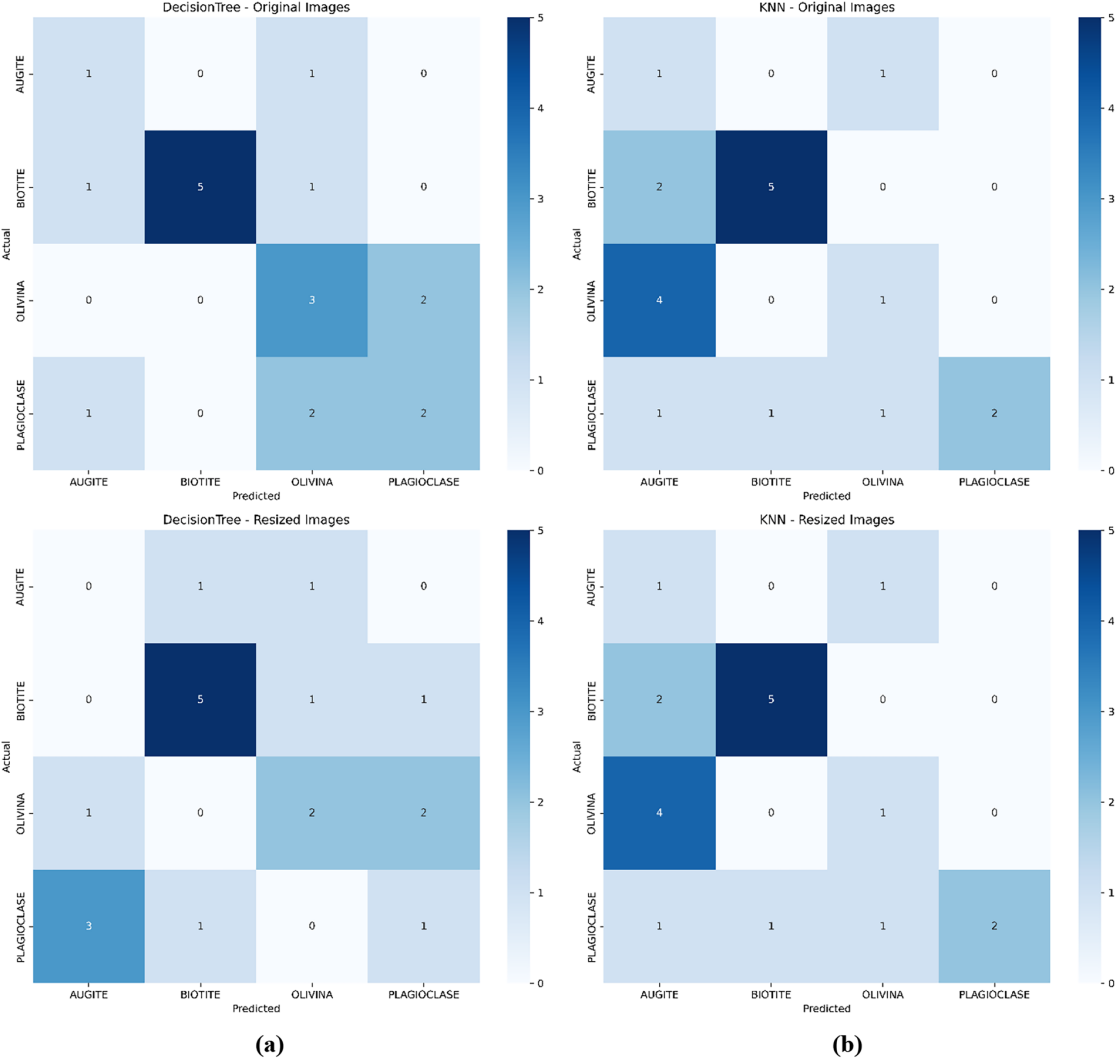
Confusion matrices for the Decision Tree (DT) and K-Nearest Neighbors (KNN) models on the Igneous and Metamorphic Dataset. **(a)** Top row presents the confusion matrices when models are evaluated using the original image dataset: Left is for the Decision Tree, and Right is for KNN. **(b)** Bottom row presents the confusion matrices when models are evaluated using the resized (150 × 150) image dataset: Left is for the Decision Tree, and Right is for KNN.

Figure 15 shows confusion matrices for the Decision Tree (DT) and K-Nearest Neighbors (KNN) models on the Igneous and Metamorphic Dataset. The top row (a) presents the confusion matrices when models are evaluated using the original image dataset, with the left matrix showing results for the Decision Tree and the right matrix for KNN. The bottom row (b) presents the confusion matrices when models are evaluated using the resized (150 × 150) image dataset, with the left matrix for the Decision Tree and the right matrix for KNN.

The confusion matrices on both datasets reveal fundamental differences in dataset quality between MUMDMC2025 and the Igneous and Metamorphic Dataset. MUMDMC2025 demonstrates exceptional class discrimination with strong diagonal dominance in both Decision Tree and KNN algorithms, achieving robust classification across all mineral categories with minimal inter-class confusion. In contrast, the Igneous and Metamorphic Dataset exhibits substantial off-diagonal scatter and poor class separation, indicating frequent misclassifications and inherent dataset limitations. The MUMDMC2025 dataset maintains consistent confusion matrix patterns across different resolutions, validating its robustness to dimensional preprocessing and suitability for practical applications. These results provide compelling evidence for the superior quality and utility of MUMDMC2025, demonstrating both high accuracy and stable performance characteristics essential for reliable geological image classification.

##### Per-Class Discrimination Analysis

**Fig. 16:**
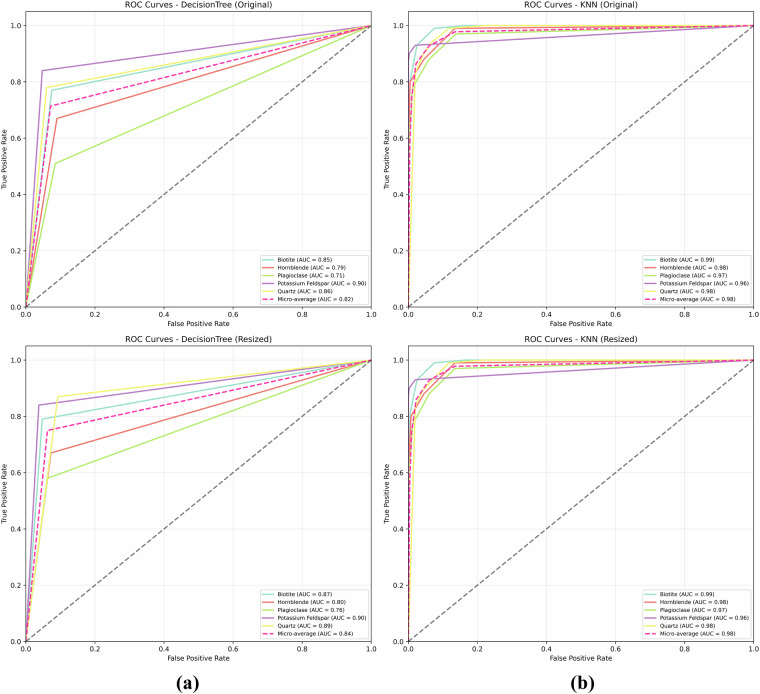
Receiver Operating Characteristic (ROC) curves for the Decision Tree (DT) and K-Nearest Neighbors (KNN) models on the MUMDMC2025 dataset. Each subplot displays the True Positive Rate (Sensitivity) against the False Positive Rate (1-Specificity) across various threshold settings. **(a)** Top row shows ROC curves for models evaluated using the original image dataset: Left is for the Decision Tree, and Right is for KNN. **(b)** Bottom row shows ROC curves for models evaluated using the resized (150 × 150) image dataset: Left is for the Decision Tree, and Right is for KNN.

Figure 16 presents Receiver Operating Characteristic (ROC) curves for the Decision Tree (DT) and K-Nearest Neighbors (KNN) models on the MUMDMC2025 dataset. Each subplot displays the True Positive Rate (Sensitivity) against the False Positive Rate (1-Specificity) across various threshold settings. The top row (a) shows ROC curves for models evaluated using the original image dataset, with the left plot for the Decision Tree and the right plot for KNN. The bottom row (b) shows ROC curves for models evaluated using the resized (150 × 150) image dataset, with the left plot for the Decision Tree and the right plot for KNN.

**Fig. 17:**
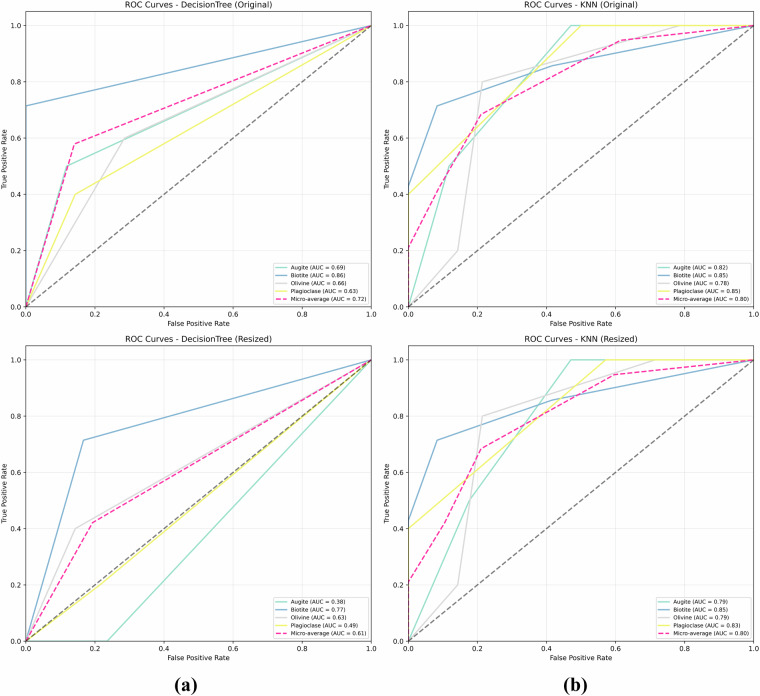
Receiver Operating Characteristic (ROC) curves for the Decision Tree (DT) and K-Nearest Neighbors (KNN) models on the Igneous and Metamorphic dataset. Each subplot displays the True Positive Rate (Sensitivity) against the False Positive Rate (1-Specificity) across various threshold settings. **(a)** Top row shows ROC curves for models evaluated using the original image dataset: Left is for the Decision Tree, and Right is for KNN. **(b)** Bottom row shows ROC curves for models evaluated using the resized (150 × 150) image dataset: Left is for the Decision Tree, and Right is for KNN.

Figure 17 presents Receiver Operating Characteristic (ROC) curves for the Decision Tree (DT) and K-Nearest Neighbors (KNN) models on the Igneous and Metamorphic dataset. Each subplot displays the True Positive Rate (Sensitivity) against the False Positive Rate (1-Specificity) across various threshold settings. The top row (a) shows ROC curves for models evaluated using the original image dataset, with the left plot for the Decision Tree and the right plot for KNN. The bottom row (b) shows ROC curves for models evaluated using the resized (150 × 150) image dataset, with the left plot for the Decision Tree and the right plot for KNN.

The ROC analysis on both datasets demonstrates fundamental differences in discriminative capabilities between MUMDMC2025 and the Igneous and Metamorphic Dataset. MUMDMC2025 exhibits exceptional per-class discrimination with consistently high AUC values above 0.85 across all mineral classes for both Decision Tree and KNN algorithms, indicating well-defined class boundaries and optimal feature representation. Conversely, the Igneous and Metamorphic Dataset shows substantial discrimination limitations with AUC values below 0.70, approaching random classification performance for several mineral classes. The MUMDMC2025 dataset maintains robust discriminative performance across different resolutions, demonstrating stability under preprocessing transformations essential for practical applications. These quantitative results validate the superior quality of MUMDMC2025 for multi-class geological classification, confirming its suitability for both research and automated geological analysis deployment.

## Methodological Validation

### Rationale Behind No Data Augmentation

The deliberate exclusion of artificial data augmentation techniques preserves authentic geological characteristics essential for reliable mineral identification. This approach maintains natural mineral textures, including complex features such as myrmekitic intergrowths and perthitic exsolution lamellae that could be distorted by artificial transformations^82^. Natural variability capture through comprehensive rotational imaging eliminates the need for synthetic augmentation while preventing overfitting^32,83^. This methodology aligns with established petrographic best practices, emphasizing authentic optical property preservation.

The MUMDMC2025 dataset represents a significant advancement in computational petrography by providing the first systematically acquired collection of mineral photomicrographs that comprehensively documents orientation-dependent optical properties across complete crystallographic rotations. This dataset addresses a critical limitation in existing petrographic databases, where incomplete optical characterization has hindered the development of robust automated mineral identification systems. The systematic imaging protocol employed—capturing 72 rotational positions at 5° increments under both plane and cross-polarized light conditions—ensures complete documentation of diagnostic optical phenomena, including pleochroism, birefringence, and extinction patterns that are fundamental to traditional petrographic analysis. The resulting 14,400 high-resolution images, equally distributed across five major rock-forming minerals from Egyptian Eastern Desert granites, provide an unprecedented level of optical detail and rotational coverage. Rigorous preprocessing techniques have standardized image quality parameters, including color balance, texture enhancement, and illumination uniformity, creating a dataset optimized for machine learning applications while preserving essential diagnostic optical characteristics. The demonstrated classification accuracy of 87.6% using K-Nearest Neighbors validates the dataset’s utility and establishes a baseline for future algorithmic developments. This comprehensive dataset fills a crucial gap in geological data resources and provides the research community with a robust foundation for developing next-generation automated petrographic analysis systems. Beyond immediate applications in mineral classification, the dataset’s systematic approach to optical documentation establishes new standards for petrographic data collection and supports broader applications in quantitative mineralogy, educational technology, and computer vision approaches to geological problem-solving.

## Usage Notes

Effective utilization of MUMDMC2025 requires systematic data preparation and model implementation protocols that leverage the dataset’s rotational imaging architecture. The following guidelines provide researchers with standardized workflows for reproducible mineral classification experiments.

### Recommended implementation workflow

#### Data preparation protocol

Download the cropped images subset (2,500 XPL images) from FigShare repository and apply pixel normalization using images = images.astype(np.float32) / 255.0 to standardize intensity values. Implement stratified train-test split (80:20 ratio) using sklearn.model_selection.train_test_split with random_state = 42 to ensure reproducible partitioning across mineral classes.

#### Model implementation standards

For baseline comparisons, employ K-Nearest Neighbors with k = 5 and Euclidean distance metric (metric = ‘euclidean’). Validate performance using 5-fold stratified cross-validation and optimize Decision Tree hyperparameters (maximum depth 5–15) through grid search implementation.

### Current dataset properties

The dataset exhibits specific constraints that users should acknowledge. Geographic scope remains limited to Egyptian Eastern Desert samples (Wadi Fatira El-beida), potentially introducing regional bias in mineral assemblage representation. The current release includes five common rock-forming minerals without altered or weathered phases. Technical constraints include a single magnification level (5 × objective), standardized resolution (150 × 150 pixels), and XPL-only public subset availability.

### Extension opportunities

Advanced methodologies can significantly enhance classification performance. Convolutional Neural Networks applied to the complete PPL/XPL dataset enable hierarchical texture analysis, while transfer learning from pre-trained Vision Transformers leverages established feature representations. Multi-modal fusion approaches combining PPL/XPL channels provide enhanced birefringence characterization capabilities.

Spatial analysis integration represents a promising research direction. Geolocation metadata can serve as additional model features for regional mineralogy prediction, while multi-scale analysis frameworks enable comprehensive petrographic interpretation.

### Reproducibility requirements

Code execution requires a Python 3.10 environment with OpenCV 4.8.0 and scikit-learn 1.2.2 dependencies. Expected performance benchmarks include KNN accuracy of 87.6% ± 1.2% and Decision Tree F1-score of 0.747 ± 0.03 for validation purposes.

## Supplementary information

Dataset 1

Dataset 1

Dataset 1A

Dataset 1B

Dataset 1C

Dataset 1D

Dataset 1E

## Data Availability

The computer code is freely available from the GitHub repository. https://github.com/MscModelTrain/MUMDMC2025-preprocessing-evaluation.git.
